# Polyhydroxybutyrate (PHB)-Based Biodegradable Polymer from *Agromyces indicus*: Enhanced Production, Characterization, and Optimization

**DOI:** 10.3390/polym14193982

**Published:** 2022-09-23

**Authors:** Mohd Adnan, Arif Jamal Siddiqui, Syed Amir Ashraf, Mejdi Snoussi, Riadh Badraoui, Mousa Alreshidi, Abdelbaset Mohamed Elasbali, Waleed Abu Al-Soud, Salem Hussain Alharethi, Manojkumar Sachidanandan, Mitesh Patel

**Affiliations:** 1 Department of Biology, College of Science, University of Hail, Hail P.O. Box 2440, Saudi Arabia; 2 Department of Clinical Nutrition, College of Applied Medical Sciences, University of Hail, Hail P.O. Box 2440, Saudi Arabia; 3 Molecular Diagnostics and Personalized Therapeutics Unit, University of Hail, Hail P.O. Box 2440, Saudi Arabia; 4 Department of Clinical Laboratory Science, College of Applied Sciences-Qurayyat, Jouf University, Sakaka P.O. Box 2014, Saudi Arabia; 5 Department of Clinical Laboratory Sciences, College of Applied Medical Sciences, Jouf University, Sakaka P.O. Box 2014, Saudi Arabia; 6 Department of Biological Science, College of Arts and Science, Najran University, Najran P.O. Box 1998, Saudi Arabia; 7 Department of Oral Radiology, College of Dentistry, University of Hail, Hail P.O. Box 2440, Saudi Arabia; 8 Department of Biotechnology, Parul Institute of Applied Sciences and Centre of Research for Development, Parul University, Vadodara 391760, India

**Keywords:** polyhyroxybutyrate, bioplastic, *Agromyces indicus*, response surface methodology, biodegradable polymer

## Abstract

Recently, there has been significant interest in bio-based degradable plastics owing to their potential as a green and sustainable alternative to synthetic plastics due to their biodegradable properties. Polyhydroxybutyrate (PHB) is a biodegradable polymer that is produced by bacteria and archaea as carbon and energy reserves. Due to its rapid degradation in natural environments, it can be considered a biodegradable plastic alternative. In the present study, a dye-based procedure was used to screen PHB-producing bacteria isolated from mangrove soil samples. Among the seven isolates, *Agromyces indicus (A. indicus)*, identified by means of 16S rRNA analysis, accumulated the highest amount of PHB. The extracted polymer was characterized by a UV–Vis spectrophotometer, Fourier-transform infrared (FTIR) spectroscopy, and for the presence of the *phbB* gene, which confirmed the structure of the polymer as PHB. The maximum PHB production by *A. indicus* was achieved after 96 h of incubation at a pH of 8.0 and 35 °C in the presence of 2% NaCl, with glucose and peptone as the carbon and nitrogen sources, respectively. The strain was found to be capable of accumulating PHB when various cheap agricultural wastes, such as rice, barley, corn, and wheat bran, were used as the carbon sources. The response surface methodology (RSM) through the central composite design (CCD) for optimizing the PHB synthesis was found to be highly efficient at augmenting the polymer yields. As a result of the optimum conditions obtained from the RSM, this strain can increase the PHB content by approximately 1.4-fold when compared with an unoptimized medium, which would substantially lower the production cost. Therefore, the isolate *A. indicus* strain B2 may be regarded as one of the best candidates for the industrial production of PHB from agricultural wastes, and it can remove the environmental concerns associated with synthetic plastic.

## 1. Introduction

In the modern world, plastics are an integral part of our daily lives, and therefore, the demand for them has increased tremendously, from 1.5 million metric tons in 1950 to approximately 370 million metric tons in 2021 [1]. Despite the low cost of these synthetic polymers, they have a negative impact on our environment, and particularly in the form of hazardous waste [2]. Because of their nondegradable nature and the fact that they cannot be recycled, plastics are the main culprits of environmental pollution. Even though a number of recycling efforts have been made, the plastics being disposed of in municipal landfills continue to cause major problems. In response, there has been an increasing number of attempts to find renewable, biodegradable, and recyclable materials (i.e., green materials that are useful for sustainability) [3]. Considering that biodegradable plastics are “eco-friendly” in nature, they offer the most feasible solution to protect the environment from petroleum-based plastic hazards. There are different types of biodegradable plastics, such as polylactic acid (PLA), *poly* (*butylene succinate*-co-butylene *adipate*) (PBSA), polycaprolactone (PCL), and poly (hydroxyalkanoates) (PHAs). These types of plastics can be used to replace synthetic plastics, such as polyethylene (PE) and polystyrene (PS), which require hundreds or thousands of years to degrade in the environment [4].

Several distinct microorganisms produce PHAs in response to stress conditions as a form of carbon storage and energy storage. In general, poly (hydroxybutyrate) (PHB) is one of the most commonly observed forms of PHAs, as it is composed of packed monomers of R-3-hydroxybutyrate (R-3-HB) [5]. PHB is the only type of polymer that is fully biodegradable in nature. Bacteria can synthesize PHB as inclusion bodies that accumulate as reserve material when their growth is subject to several different stress conditions [6]. This polymer exhibits properties that are similar to several synthetic thermoplastics, including polypropylene. The advantages of these types of biodegradable plastics are that they are useful for extensive applications and can be produced on a mass scale. This will enable us to replace the petroleum-based plastics that are currently used by the industry [7].

The high production costs of PHB in comparison with plastics derived from petrochemicals are one of the major problems for the extensive production and commercialization of this product. A great deal of effort has been put into reducing the cost of producing PHB in recent years by utilizing strategies such as developing efficient bacterial strains and optimizing the fermentation and recovery processes [8,9]. It has been suggested that, based on most of the reports regarding the production of PHB, the carbon substrate cost is one of the major contributors to the overall production costs of PHB [10]. It is for this reason that the choice of an efficient carbon substrate is one of the most important steps in determining the final cost of the product. Alternatively, a renewable, economically feasible, and most readily available carbon substrate can be selected and used for both microbial growth and the efficient production of PHB by microorganisms [8,10]. Therefore, this study was designed to isolate and screen PHB-producing bacteria from mangrove soil samples with a focus on enhanced biopolymer production, including characterization and optimization.

## 2. Materials and Methods

### 2.1. Sample Collection and Isolation of Bacteria

A sterilized falcon tube was used to collect the mangrove soil samples and was taken to the laboratory. Serial dilutions of the collected sample were performed, and 0.1 mL of each dilution was spread and plated on Zobell marine agar (peptone: 5.000 g/L; yeast extract: 1.000 g/L; ferric citrate: 0.100 g/L; sodium chloride: 19.450 g/L; magnesium chloride: 8.800 g/L; sodium sulfate: 3.240 g/L; calcium chloride: 1.800 g/L; potassium chloride: 0.550 g/L; sodium bicarbonate: 0.160 g/L; potassium bromide: 0.080 g/L; strontium chloride: 0.034 g/L; boric acid: 0.022 g/L; sodium silicate: 0.004 g/L; sodium fluorate: 0.0024 g/L; ammonium nitrate: 0.0016 g/L; disodium phosphate: 0.008 g/L; agar: 15.000 g/L; pH: 7.6 ± 0.2) with 1% glucose [11]. Using a shaker incubator, cultures were incubated at 35 °C for 72 h at 150 rpm, and purified colonies were stored at 4 °C. The isolated strains were purified and maintained on Zobell marine agar slant at 4 °C.

### 2.2. Screening the Isolates for PHB Production

#### Screening by the Sudan Black B Method

In order to detect the presence of PHB granules in the isolated strains, Sudan Black B dye was used as the primary screening method. A Zobell marine agar supplemented with 1% glucose supplement was added to the nutrient agar medium. The isolated bacterial strain was spread on the Zobell marine agar supplemented with 1% glucose plates, and was allowed to incubate for 24 h at 30 °C. The Sudan Black B stain was made by dissolving 0.02 g of powder in 100 mL of 70% ethanol. Upon the completion of incubation, Sudan Black B dye was spread over the plates and was allowed to stand for 30 min before being removed. To remove the excess stain, plates were washed with 96% ethanol and left to dry. It has been observed that colonies unable to incorporate the Sudan Black B dye appear white, whereas colonies producing PHB appear bluish-black [12].

### 2.3. Screening by Nile Blue A Method

To screen the isolates that had positive results for PHB, the Nile Blue A staining technique was further used [13]. Bacterial isolates were grown in 10 mL of Zobell marine agar supplemented with 1% glucose as the sole carbon source, and incubated with shaking at 120 rpm for 72 h at 37 °C. As bacterial cultures were grown, they were centrifuged at 6000× *g* rpm for 10 min at 4 °C. Using 1 mL of sterile distilled water, the cell pellets were resuspended. We then proceeded to fix smears of these cell suspensions to glass slides and stain them with 1% Nile Blue A (Hi-Media^®^, Mumbai, India), and they were observed under the fluorescence microscope. PHB accumulation was determined from the appearance of orange granules within the cells, which was regarded as a positive signal.

### 2.4. Molecular Identification of PHB-Accumulating Bacteria by 16S rRNA Gene Analysis

Genomic DNA was extracted from the bacteria using a bacterial genomic DNA isolation kit (GenElute^TM^, Shanghai, China, Sigma-Aldrich^®^, Bangalore, India). As described by Sambrook, Fritch, and Maniatis (1982) [14], the optical density (OD) of the isolated genomic DNA was measured at 260 and 280 nm (UV-1800, Shimadzu Spectrophotometer, Kyoto, Japan). By the electrophoresis of the extracted genomic DNA in agarose gel (0.8%), the purity of the extracted genomic DNA was further checked. The amplification of universal primers 27F (5′AGAGTTTGATCMTGGCTCAG3′) and 1492R (5′CGGTTACCTTGTTACGACTT3′) was carried out using a final volume of 20 μL, containing 1× ReadyMix™ Taq PCR reaction mix (Sigma^®^, Bangalore, India), 10 pmol of each primer, ~10 ng of genomic DNA template, and nuclease-free water to make up the volume [15]. In order to carry out the reaction, we used a thermal cycler (Applied Biosystems Veriti^®^, Waltham, MA, USA). The PCR cycling conditions were as follows: 95 °C for 5 min, 30 cycles of 95 °C for 1 min, 55 °C for 1 min, and 72 °C for 1 min, as well as a final extension step at 72 °C for 10 min. The PCR products that were amplified were detected on an agarose gel (1%), after electrophoresis staining with ethidium bromide and visualization under UV light, of the gel documentation system from Bio-Rad^®^ (Hercules, CA, USA). Following successful amplifications, PCR products were purified using a GenElute^TM^ PCR clean-up kit, which was subsequently sequenced by Eurofins Genomics India Pvt Ltd., Bangalore, India. The sequence analysis was performed using sequencing analysis software version 5.4 (Applied Biosystems^®^, Waltham, MA, USA), as well as BioEdit 7.2.5 (North Carolina State University, Raleigh, NC, USA). A sequence-match analysis was performed on the sequences with the NCBI’s Basic Local Alignment Search Tool (BLAST). The sequences were submitted to the NCBI GenBank database.

### 2.5. Amplification of phbB Gene in Potent Isolates

The amplification of the *phbB* gene in the isolate was carried out via utilizing a primer set of phbBF-5′-ATGAGCAATCAACGAATTGCA-3′ and phbBR-5′-TCATTGCATGTTCAGACCGC-3′ [16]. The amplification was carried out in a thermal cycler (Applied Biosystems Veriti^®^, Waltham, MA, USA) using 20 μL of the final volume. The PCR mixture contained 1× ReadyMix™ Taq PCR reaction mix (Sigma^®^, India), 10 pmol of each primer, ~10 ng of genomic DNA template, and nuclease-free water to make up the volume. The PCR cycling conditions were as follows: 95 °C for 5 min, 35 cycles of 95 °C for 2 min, 60 °C for 30 s, 72 °C for 2 min, a final extension step at 72 °C for 10 min, and an indefinite hold at 4 °C. The PCR product was then electrophoretically separated on a 1% agarose gel, and the banding pattern was then viewed under UV light using a gel documentation system from Bio-Rad^®^ (USA).

### 2.6. Production, Detection, and Extraction of PHB

The PHB-producing bacterial isolate was inoculated in 10 mL of sterile Zobell marine broth and incubated at 37 °C for 24 h. Upon completion of incubation, 10% (*v*/*v*) of the culture was transferred aseptically into a 250 mL conical flask containing 100 mL of sterile Zobell marine broth, and it was incubated for 72 h at 37 °C and 120 rpm [17]. As a next step, the culture broth was centrifuged at 5000× *g* rpm for 15 min, the supernatant was discarded, and the pellets were dried. In order to carry out cell lysis, 10 mL of sodium hypochlorite solution was added to the dried pellets and incubated at 50 °C for 2 h. Thereafter, the mixture was centrifuged again for 15 min at 5000× *g* rpm, the supernatant was discarded, and the pellets were subsequently washed with distilled water, acetone, and methanol. As a final step, they were dissolved in 5 mL of boiling chloroform. By using Whatman no. 1 filter paper, the non-PHB cell matter was removed from the samples by filtration. Following the evaporation of the chloroform, the obtained PHB was stored for further analysis [18].

### 2.7. Quantitative Analysis of PHB

In order to grow the bacterial culture, the procedure described above was followed. After collecting the cell pellets, they were dried overnight in a vacuum-drying oven at 60 °C to estimate the dry weight (g/L). In order to estimate the percentage of intracellular PHB accumulation, the percentage composition of PHB in the dry cell weight was taken into account [19].

The PHB accumulation (%) was calculated as follows: dry weight of extracted PHB (g/L)/dry cell weight (DCW) (g/L) × 100

### 2.8. Characterization of PHB

In order to characterize the extracted PHB, ultraviolet–visible (UV–Vis) spectroscopy and Fourier-transform infrared (FTIR) spectroscopy analyses were performed. As a first step, UV–Vis spectrophotometer analysis was performed, for which extracted PHB was dissolved in chloroform and scanned in the range of 200–320 nm (UV–Vis spectrophotometer, Shimadzu, Kyoto, Japan). The FTIR (Bruker^®^, Billerica, MA, USA) spectrum was recorded in the wave range from 400 to 4000 cm^−1^ [20].

### 2.9. Optimization of PHB Production

In the process of optimizing the PHB production, various factors were considered, including incubation periods (24–144 h), pH values (6.5–9.0), incubation temperatures (25–45 °C), NaCl concentrations (2–10%), carbon sources (glucose, fructose, sucrose, maltose, and arabinose), and nitrogen sources (peptone, yeast extract, glycine, urea, and yeast extract + peptone). For each parameter, fermentation was carried out in a 250 mL Erlenmeyer flask containing 50 mL of Zobell marine broth, with respective conditions, and incubated under shaking conditions. According to the previous description, the PHB production was estimated. In order to determine the cell dry bulk weight (biomass), the cell pellets were centrifuged at 6000× *g* rpm for 10 min, dried at 60 °C, and then expressed as grams per liter after centrifugation (6000× *g* rpm/10 min). The experiments were conducted in triplicate [21].

### 2.10. Optimization of PHB Production by Response Surface Methodology

Using the classical one-factor-at-a-time (OFAT) method, the most crucial independent variables (rice bran, peptone, and temperature) were optimized using the statistical technique known as the central composite design (CCD) to increase the PHB production while keeping the other variables constant. As shown in Table 1, each independent variable in the design matrix was investigated at five different levels (−α; −1; 0; +1; +α), with an α-value ± 1.6817. A total of 20 experiments were run, with six replicates of the central points, using Design Expert^®^ (Stat-Ease, Minneapolis, MN, USA) software version 12.0 (Table 2). The coefficient R^2^ was used to express the quality of the fit for the quadratic equation, and the F-test was used to determine its statistical significance. A *t*-test was used to determine the significance of each variable’s effect on the PHB production. The CCD result was analyzed using analysis of variance (ANOVA). The data were interpreted to produce a response surface in the form of contours and 3D plots illustrating the interactions of the factors.

### 2.11. Statistical Analysis

Each experiment was performed in triplicate, and the results were expressed as means ± SDs. GraphPad Prism 5.0 (GraphPad Software, Inc., San Diego, CA, USA) was used for the analyses.

## 3. Results

### 3.1. Isolation, Screening, and Selection of PHB-Producing Bacteria

**Figure 1:**
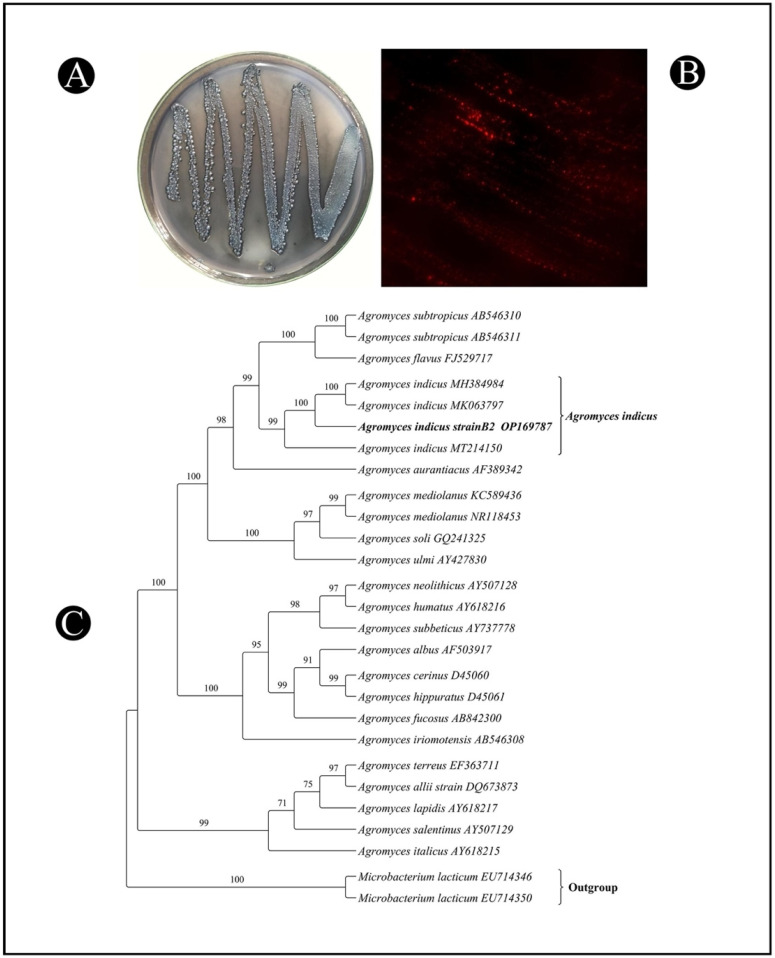
Screening of promising bacterial isolate (B2) from the mangrove soil samples for PHB production: (**A**) plate staining with Sudan Black B; (**B**) slide staining with Nile Blue A, as observed under fluorescence microscope; (**C**) phylogenetic tree based on 16S rRNA nucleotide sequences of the bacterial isolate *A. indicus* strain B2, with other sequences of published *Agromyces* strains generated by the neighbor-joining method. The numbers at the nodes indicate the levels of bootstrap support (%) based on 1000 resampled datasets.

In the present study, a total of seven different bacterial isolates were obtained from the collected mangrove soil samples. Initially, the bacterial isolates were screened with Sudan Black B via the colony-staining method as the primary method of screening. As a result, only three of the seven bacterial isolates were capable of accumulating the PHB granules. In the second screening, Nile Blue A staining was performed under a fluorescent microscope. It can be seen from Figure 1A,B that the dark blue colonies were stained with Sudan Black B, and orange-colored granules were observed inside the bacterial cells, which was taken as a positive indication that PHB was being produced by the bacteria. It was found that one isolate, B2, had a high ability to produce PHB, which led to it being selected for further investigation.

### 3.2. Identification of High-PHB-Producing Bacterial Isolate Using the 16S rRNA Gene and Its Phylogenetic Analysis

The genomic DNA was extracted from the PHB-producing isolate B2 and was used for the PCR amplification of its 16S rRNA gene. Through the amplification and sequencing of the 16S rRNA gene, it was revealed that the isolate B2 shared 99.71% identity with the *Agromyces indicus* strain Mix6. The gene sequence obtained from the isolate B2 was submitted to GenBank with the accession number OP169787. The phylogenetic tree of the isolate was constructed in order to determine its relationship with the other species of the genus *Agromyces* (Figure 1C).

### 3.3. Amplification of phbB Gene

**Figure 2:**
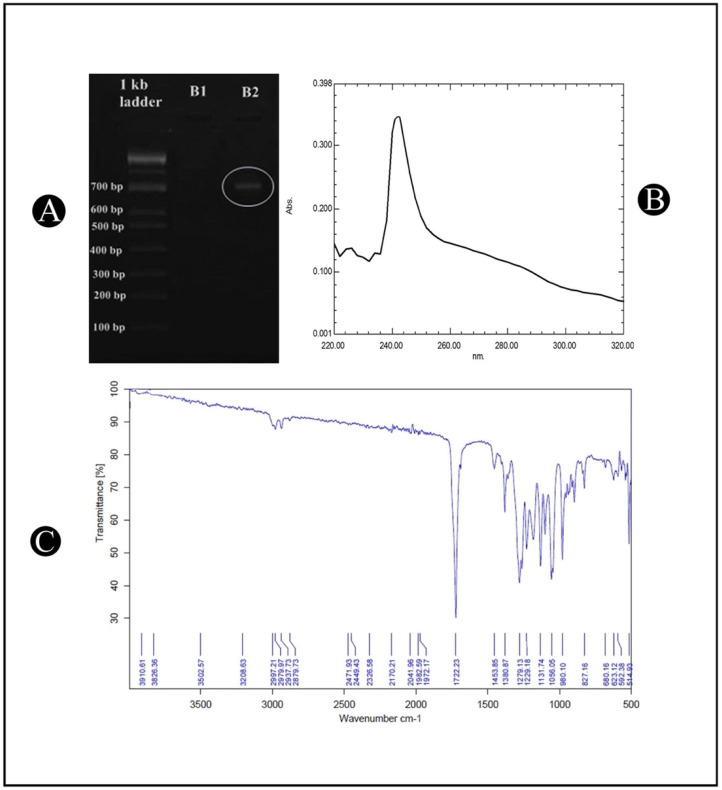
(**A**) Amplification of *phbB* gene in *A. indicus* strain B2 by PCR amplification followed by agarose gel electrophoresis; (**B**) UV–Vis-spectrophotometer-scanning spectrum of extracted PHB from *A. indicus* strain B2; (**C**) FTIR analysis of extracted PHB from *A. indicus* strain B2.

The PCR amplification of the *phbB* gene was performed to characterize the *A. indicus* B2 strain for the production of PHB. A PHB synthase is an essential enzyme in the biosynthetic pathway of PHB, and it is encoded by this gene. As a result, in *A. indicus* B2, the *phbB* gene was present. The clear distinct banding pattern of around 700 bp in the *A. indicus* B2 strain confirms the presence of *phbB* (Figure 2A).

### 3.4. Characterization of PHB

In order to confirm the structure of PHB, the extracted polymer was characterized by UV–Vis spectrophotometers and FTIR analysis. The use of UV–Vis spectroscopy for detecting PHB is a recognized and widely used method for determining its presence in the environment. A spectroscopic analysis of the extracted PHB showed a clearly symmetric absorption spectrum, with the peak maximum at 241 nm (Figure 2B). The analysis of the PHB produced by the *A. indicus* B2 strain was performed using FTIR spectroscopy in order to identify the different functional groups that are present in PHB. The FTIR spectrum of the PHB, which was recorded between 4000 and 400 cm^−1^ (Figure 2C), showed a sharp absorption band at 1722.23 cm^−1^, which corresponds to the carbonyl (C=O) stretching of the ester, and another band at 1279.13 cm^−1^, which corresponds to the –CH group. These bands have been reported and labeled as markers of PHB. A series of bands between 1000 and 1300 cm^−1^ showed the stretching of the C–O bond of the ester group. The bands at 2979.97 and 2937.73 cm^−1^ indicated the presence of the methyl (CH_3_) and methylene (CH_2_) asymmetric and symmetric stretching modes, respectively. Additionally, the bands of minor relevance at 3502.57 cm^−1^ are related to a terminal OH group.

### 3.5. Effect of Incubation Time on PHB Production

**Figure 3:**
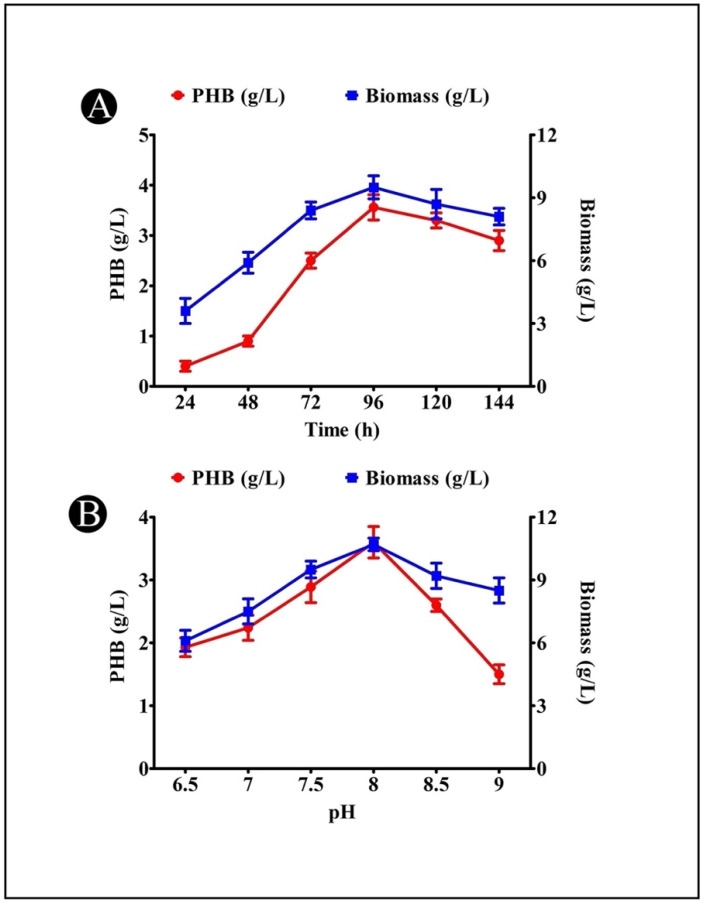
(**A**) Effect of incubation time on PHB production by *A. indicus* strain B2; (**B**) effect of pH on PHB production by *A. indicus* strain B2.

It was found that the incubation time had a significant effect on the amount of PHB produced by the *A. indicus* B2 strain. The PHB production was found to increase when the incubation time was increased to 96 h (3.54 g/L), but after that, the production of PHB decreased as the incubation time increased (Figure 3A). The decrease in the PHB production indicated that the bacteria used PHB as a nutrient source, which resulted in unsuitable growing conditions due to the fact that the nitrogen and carbon sources were insufficient in the medium at the time.

### 3.6. Effect of Medium pH on PHB Production

In order to determine the optimum pH level for the production of PHB, a variety of pH levels (6.5, 7.0, 7.5, 8.0, 8.5, and 9.0) were tested. The maximum PHB production was found at a pH of 8.0 (3.62 g/L) by the *A. indicus* B2 strain, as shown in Figure 3B. A low amount of PHB was produced at pH values of 6.0 and 9.0. Based on the results of this experiment, it was revealed that slightly alkaline conditions were suitable for generating high levels of PHB.

### 3.7. Effect of Temperature on PHB Production

**Figure 4:**
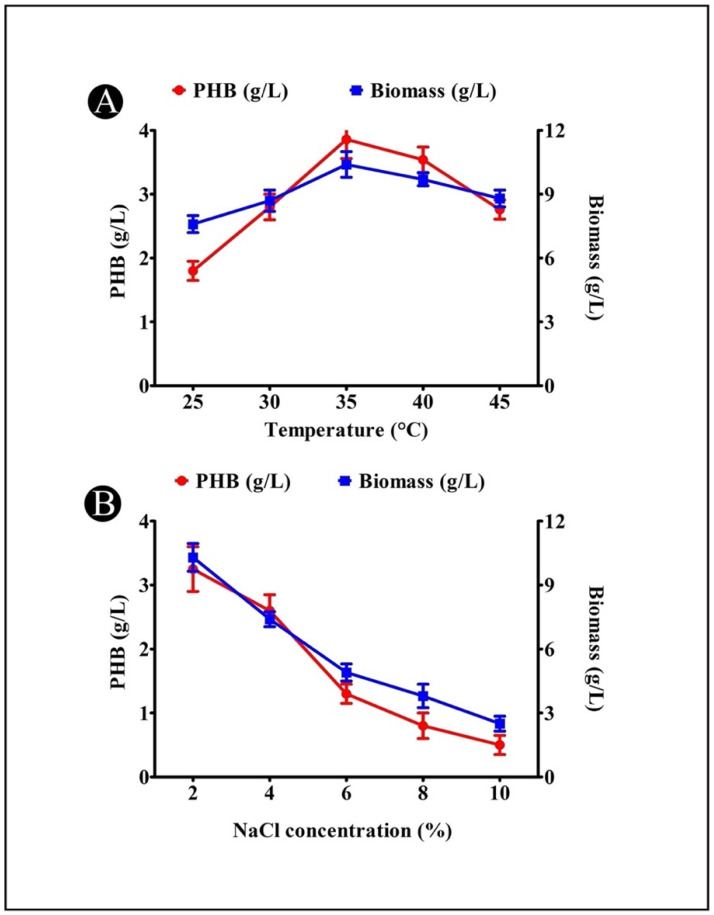
(**A**) Effect of incubation temperature on PHB production by *A. indicus* strain B2; (**B**) effect of NaCl concentration on PHB production by *A. indicus* strain B2.

An incubation period at various temperatures was conducted with the *A. indicus* B2 strain in production media. The maximum amount of PHB was observed to be produced at a temperature of 35 °C (3.86 g/L) (Figure 4A). The variation in the PHB production at different temperatures can be explained by the fact that temperatures other than the optimal ones had an impact on the enzymes responsible for synthesizing PHB, thereby reducing the activity of the enzymes.

### 3.8. Effect of NaCl Concentration on PHB Production

There was a maximum production of PHB at 2% (*w*/*v*) NaCl, reaching a maximum of 3.25 g/L (Figure 4B). It is evident from this result that the isolate belongs to a group of mild halophiles, which is partially proportional to the salinity recorded in the mangrove samples tested. Based on this result, it is evident that it is essential to control the salinity of the medium within a reasonable range in order to prevent high osmotic stress exerting an effect on the PHB production, and particularly as increasing the salt to 6% results in a decrease in the PHB production.

### 3.9. Effects of Different Carbon Sources on PHB Production

**Figure 5:**
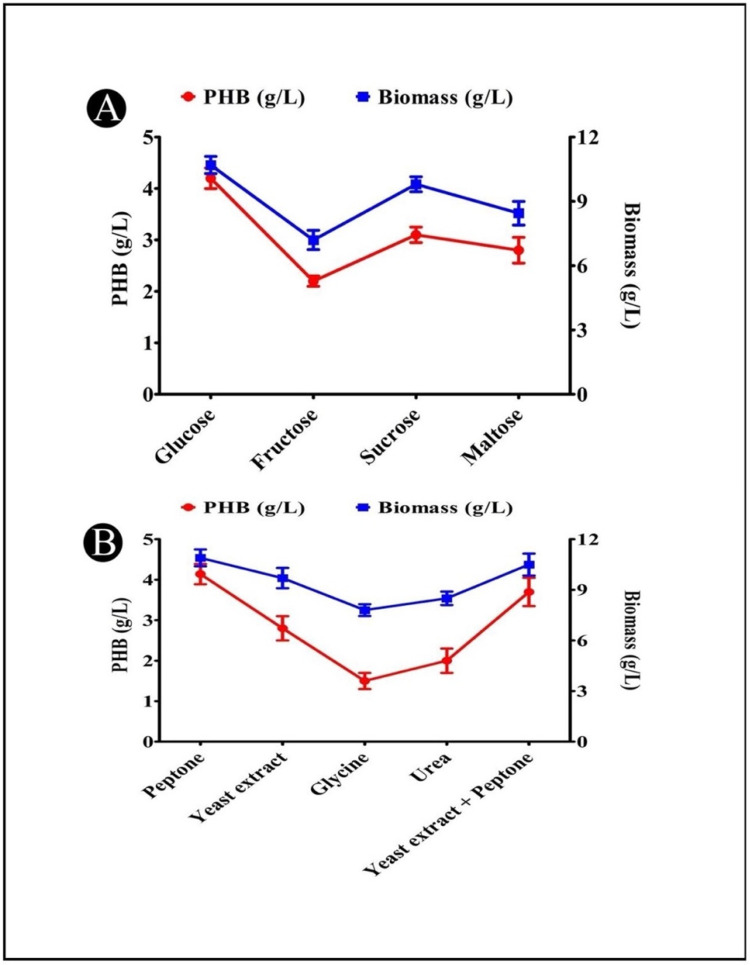
(**A**) Effects of different carbon sources on PHB production by *A. indicus* strain B2; (**B**) effects of different nitrogen sources on PHB production by *A. indicus* strain B2.

The effects of different carbon sources (glucose, fructose, sucrose, maltose, and arabinose) on the PHB production in the cultures are represented in Figure 5A. According to the results of this experiment, the ability of the bacteria to utilize different carbon sources was variable. This ability might be influenced by a number of factors, such as the kind of substrates used, and the types of enzymes produced by the bacteria. The PHB production was most efficient when glucose (4.20 g/L) was the carbon source, followed by sucrose and maltose as the next most efficient carbon sources. Bacteria produce more PHB when fed with glucose, which is an easily digestible carbon source that is readily available.

### 3.10. Effects of Different Nitrogen Sources on PHB Production

A comparison of the effects of different nitrogen sources (peptone, yeast extract, glycine, urea, and yeast extract + peptone) on the production of PHB is presented in Figure 5B. The *A. indicus* B2 strain was able to produce the maximum amount of PHB when grown in production media that had peptone (4.14 g/L) added to it. Most of the bacteria were found to produce PHB at a higher rate if complex nitrogen sources, such as peptone, were used, while other nitrogen sources were found to significantly reduce the PHB production at a higher rate. There is a possibility that this was due to the relatively low nitrogen content of peptone, which, in turn, favored a higher accumulation of PHB.

### 3.11. Production of PHB from Different Agricultural Wastes

**Figure 6:**
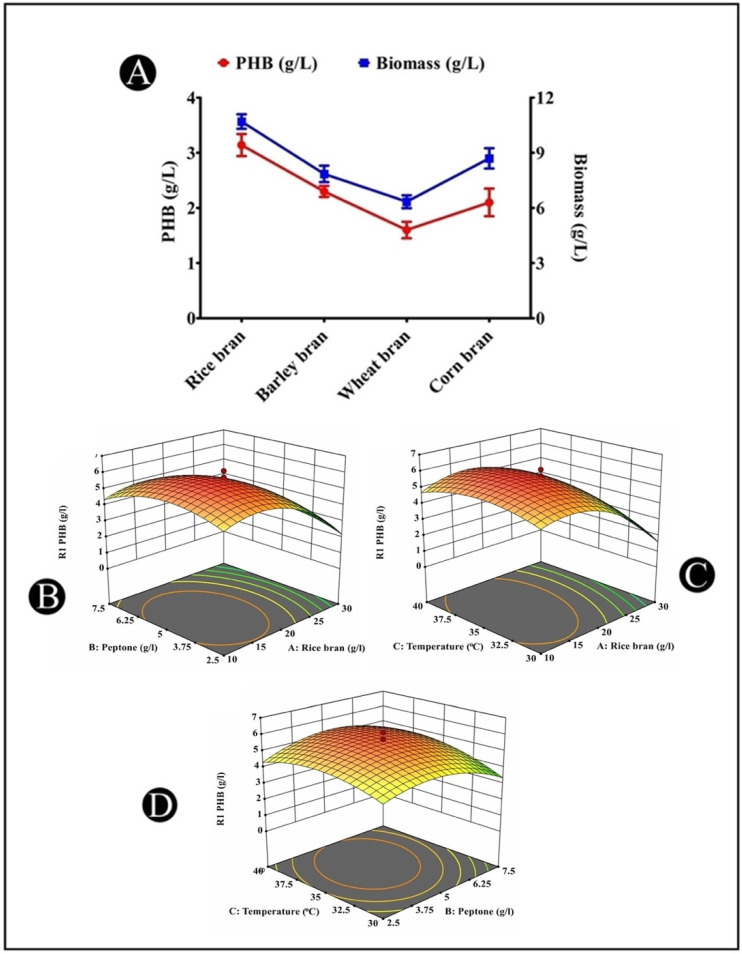
(**A**) Effects of different cheap agricultural wastes on the PHB production by *A. indicus* strain B2; (**B**–**D**) 3D surface plots representing PHB production from culture broth of *A. indicus* strain B2 as affected by cultural conditions: (**B**) rice bran and peptone; (**C**) rice bran and temperature; (**D**) peptone and temperature. The red dots indicate the optimal values of the variables taken in the study at which the highest accumulation of PHB was observed at that particular point.

There is an abundant availability of wastes generated in the agricultural sector, and these wastes include rich sources of carbohydrates. Many bacterial species have the ability to utilize these diverse and cheap carbon wastes because they possess hydrolytic enzymes that are capable of metabolizing these complex residues. Different agricultural waste materials (rice bran, barley bran, corn bran, and wheat bran) were employed as the main carbon sources for the PHB production by the *A. indicus* B2 strain, among which rice bran had the highest PHB production (3.14 g/L) (Figure 6A).

### 3.12. Optimization of PHB Accumulation by Response Surface Methodology

In this investigation, the CCD was employed to study the interactions among the three independent variables (rice bran, peptone, and temperature) to determine their optimal levels for PHB accumulation. The full experimental matrix with respect to their actual and predicted responses is represented in Table 2. In each trial, the response values (Y = PHB production) were the average of the triplicates. The *A. indicus* strain B2 exhibited significant differences in PHB production, which shows the apparent influence of the process variables within their range (Table 2). The medium containing 20 g/L of rice bran, 5 g/L of peptone, and with a temperature of 35 °C produced the most PHB (6.11 g/L), while the medium containing 36.81 g/L of rice bran, 5 g/L of peptone, and with a temperature of 35 °C produced the least (0.01 g/L). The following quadratic equation was fitted to determine the effect of each independent variable on the response:

$$Y=+5.65-1.21\;A-0.1427\;B+0.3317\;C+0.0687\;\mathrm{AB}+0.3837\;\mathrm{AC}+0.2787\;\mathrm{BC}\;-2.44{\;A}^{2}-0.8603{\;B}^{2}-0.6641{\;C}^{2}$$

where Y is the predicted response, and A, B, and C are the coded values of the test variables: rice bran (g/L), peptone (g/L), and temperature (°C), respectively.

An ANOVA was applied for the analyses of the regression coefficient, prediction equations, and case statistics. The model’s adequacy was assessed using an ANOVA, which was validated using Fisher’s statistical analysis, and the results are shown in Table 3. The model F-value and *p*-value were 31.37 and <0.0001, respectively, indicating that the model was significant. The “Lack of Fit” of the F-value was 4.97, indicating that the “Lack of Fit” was also not significant in comparison with the pure error. A ”Lack of Fit F-value” had a 5.15% chance of occurring due to noise. The “Pred R-Squared” of 0.7742 is consistent with the “Adj R-Squared” of 0.935.

The response surface contour plots for the PHB production/accumulation generated by the predicted model are displayed in a 3D graph (Figure 6B–D). According to the statistics, the PHB production/accumulation initially increased significantly up to a certain level, and then the production/accumulation was gradually reduced with its higher value. The diagnostic plots showed that the model satisfied the analysis-of-variance assumptions and also reflected the accuracy and applicability of the RSM for the optimization of the process for PHB production/accumulation.

## 4. Discussion

Plastic is among the most major pollutants that are being produced, causing global environmental pollution. Thus, there is a need to develop an alternative to replace this non-biodegradable pollutant, which is used by everyone on a daily basis for a variety of purposes [22]. In spite of the fact that the idea of producing and extracting biodegradable plastic was developed many years ago, some modifications need to be made before it can be used on a large scale in industries to replace the plastic that is derived from petroleum [23]. Due to the high cost of producing bioplastics, a number of techniques have been used to produce them on a large scale. However, all the researchers who are actively involved in this field should focus on the selection of a proper strain of bacteria that is capable of producing or accumulating PHB at a high level. The use of terrestrial bacteria that are capable of producing bioplastics has been extensively studied [24]; however, the mangrove environment has received the least attention compared with its terrestrial counterparts [25]. Therefore, the purpose of this study was to isolate the bacteria that can produce PHB from soil samples collected from a mangrove area.

A dye-based procedure was used to detect the intracellular PHB in the isolated bacteria, in which black- and orange-colored lipid granules were considered as indicators of PHB accumulation by Sudan Black B and Nile Blue A staining, respectively [26]. As the lipid granules are comprised of component parts that are heavily incorporated into the cellular structure, the high susceptibility of the granules to staining with Sudan Black B and Nile Blue A has been attributed to this incorporation [27]. Moreover, it is easier to distinguish between the cell contents at different wavelengths [28]. Based on the findings of this study, it appears that the habitats of mangrove areas are potential sources of bacteria that can produce PHB. It is thought that the absence of river inflows and low sediment and nutrient levels in the mangrove environment results in the accumulation of PHB by microbial communities as a survival mechanism [29].

An accurate method of identifying bacteria is to use the 16S rRNA gene. This has been proven to be a reliable modern method that has replaced the traditional method of identifying bacteria by their phenotypic characteristics [30]. Prokaryotes possess a 16S rRNA subunit, which is a component of the 30S ribosomal subunit. A genetic marker called 16S rDNA encodes this gene [31]. On the basis of the 16S RNA gene, an isolated B2 strain was identified as the *A. indicus* B2 strain, which is the most active isolate accumulating PHB granules. Different species of *Agromyces* are found in marine and mangrove environments that are capable of producing biopolymers. By analyzing 16S rRNA sequences, other strains of bacteria producing high amounts of PHB have been identified from mangrove environments, such as *Bacillus thuringiensis* and *Erythrobacter aquimaris* [21,31].

PHB is produced and synthesized by bacteria using three regulatory enzymes: β-ketothiolase, acetoacetyl-CoA reductase, and PHA synthase/polymerase. These enzymes are coded by *phbA, phbB*, and *phbC*, respectively [32]. Among them, *phbC* is present at the upstream from *phbA–phbB*. D(-)-3-hydroxybutyryl-CoA is formed when the reductase interacts with either enoyl-CoA hydratase or epimerase. The presence of *phbB* in the *A. indicus* B2 strain indicates the presence of a *phb* gene cluster that mediates the PHB production. The production of PHB is mediated through many different genetic mechanisms, but the majority of the production is mediated through the *phbB* gene cluster in bacteria, as demonstrated in *Rhodobacter sphaeroides*, [33], *Azotobacter* sp. [34], and *Azospirillum brasilense* [35]. Many bacteria exhibit poor growth and less PHB accumulation in *phbB* mutants, which suggests that it may play a role in the process of PHB synthesis [36]. In addition, the amplification of the *phbB* gene in isolates has been carried out in many previous studies to determine the molecular nature of the PHB synthesis in them [34]. Accordingly, to ensure that *phbB* is involved in PHB synthesis, its amplification was performed in the isolates.

The production of PHB by environmental isolates is affected by a number of factors, including carbon, nitrogen, temperature, pH, the incubation time, the salt concentration, etc. In the present study, the production of PHB was increased gradually over the first 24 h during the logarithmic phase of the growth of the *A. indicus* B2 strain. When the growth reached the stationary phase after 96 h, a result of 3.54 g/L was obtained. A direct correlation was observed between bacterial growth and PHB accumulation, as reported by Gomaa [37], who reported that 96 h was the optimum cultivation time for *B. subtilis* and *E. coli*. The amount of PHB that is accumulated by a particular species of bacteria is directly related to its biomass. As the biomass of a specific species of bacteria is reduced by the depletion of carbon, the PHB accumulation is inevitably reduced, making the PHB more readily available for consumption [38]. The literature has documented several periods of cultivation that have been found to be optimal for the bacterial accumulation of PHB. As an example, 48 h of incubation was found to be optimal for *Rhizobium alti*, *Pseudomonas stutzeri*, and *Bacillus* sp. [38,39], and incubation times of 40 h for *B. subtilis*, an isolate from sponges [40], and 24 h for the halophilic *B. megaterium* were found to be optimal [41]. Meanwhile, it was previously reported that the production of PHB decreased following the end of the ideal fermentation period [42,43]. According to the study, PHB was reported to accumulate as a food reserve by marine bacteria, such as *Bacillus* sp. CS-605, *Bacillus megaterium*, and *Vibrio harveyi*, which was depolymerase-degraded once the external carbon sources were exhausted at the stationary phases, thereby promoting the survival of the bacteria.

There is evidence that PHB accumulation is influenced by the pH of the solution because of its effect on the bioavailability of the trace elements [43], as well as the enzymes involved in the synthesis of PHB: β-ketothiolase, acetoacetyl-CoA reductase, and PHA polymerase [13]. It was found that a pH of 8.0 was the optimal pH for the accumulation of PHB (3.62 g/L). The results of the present study are similar to the results reported by Kalaivani and Sukumaran [26], in which they found that *Saccharococcus thermophilus* produced the most PHB under alkaline conditions (pH 8.0). As well, it has been reported that the production of PHB by *V. harveyi* 284 was also higher under alkaline conditions (pH 8.0). It was observed that the production of PHB declined at a pH of 9.0, and at a pH of 6.5 when the reaction was carried out. The accumulation of PHB was supported in a pH range of 7–8.5, but it was significantly reduced when the pH range exceeded this threshold [44].

As far as the effects of temperature on the variation in PHB production are concerned, this can be explained by the fact that temperatures other than the optimal one has been found to reduce the activity of the enzymes responsible for the synthesis of PHB [2]. As a result of this study, it appears that there is a significant relationship between PHB production and the incubation temperature. In agreement with other studies [32,34], the maximum production of PHB and bacterial biomass occurred at a temperature of 35 °C, indicating that this was the optimal temperature. The PHB production was affected differently by temperature in different genera based on their morphologies. It was shown that a temperature of 30 °C was the best temperature for producing PHB by *Nacardiopsis potens*, *Rhizobium elti*, *P. stutzeri*, and *V. harveyi* [33,37], and a temperature of 50 °C was the best for producing PHB by marine *S. thermophilus* [28].

It is evident that the salt concentration plays a pivotal role in bacterial growth, as well as the production of biopolymers [44]. There was a maximum level of PHB production at 2% (*w*/*v*) NaCl. The results indicated that this isolate has a slight halophilic phenotype. In the mangrove environment, the majority of microbes are mild-to-moderate halophiles. Based on the results of the present study, it seems that the salinity of the medium must be controlled within a range that prevents high osmotic stress from having an adverse effect on the PHB production [42], as an increase in the salt concentration to 6% results in a decrease in the PHB production. A variety of salt concentrations were found to affect the accumulation of PHB in the bacterial strains, such as 5% for *V. proteolyticus* [45], 2.5% for *Nacardiopsis potens* [46], 3% for *Bacillus* sp. CS-605 [13], 2% for *V. harveyi* [43], and 1.5% for *Vibrio azureus* [44].

In the production of PHB, the carbon source is one of the major cost-associated factors [47]. It is imperative to note that carbon is one of the most important nutrients that plays an influential role in the production of PHB. This is because the bacteria store carbon in the form of PHB granules. Based on the results of the study, it can be concluded that glucose was the preferred carbon source for the production of PHB. A number of bacteria, such as *Vibrio azureus* BTKB33, *Bacillus megaterium*, *Bacillus* sp., and *Alcaligens eutrophus*, showed that it is an effective carbon source for the growth and production of PHB [2,48].

Apart from the carbon sources, it has been shown that the bacterial biomass and the amount of PHB produced can be affected by different organic and inorganic nitrogen sources. As a matter of fact, the nitrogen source represents the most significant limiting factor for PHB production. This is because the strains accumulate lipid granules as a way of surviving under unbalanced nutritional conditions [49]. The bacteria require a high concentration of nitrogen at the beginning of the fermentation process in order to produce a large amount of biomass. Upon the depletion of nitrogen, the bacteria begin to produce polymers as a survival mechanism [49]. Compared with the initial productivity obtained using peptone as the nitrogen source, the PHB productivity obtained using peptone was superior, which may be due to the fact that it is an excellent nitrogen source and serves as the precursor for amino acids and growth factors [50]. However, the possibility exists that there may be more than one mechanism underlying the accumulation of PHB among the various microbes. This is borne out by the fact that ammonium sulfate was found optimal for the production of PHB by *B. sacchari* [46], *R. etli* E1, and *P. stutzeri* [51].

Carbon and nitrogen sources, as well as environmental factors, such as the incubation time, temperature, and initial pH, all play important roles in bacterial fermentation [52], as these have been shown to influence the PHB production by other microorganisms [53,54]. A flask-scale RSM employing the CCD was used to enhance PHB production/accumulation. When using the RSM-optimized medium, the highest 6.1 g/L PHB production was achieved, which was increased ~1.4-fold over the initial unoptimized conditions. The RSM has previously been noted as a highly effective tool to enhance the PHB production by various microorganisms.

In a newly engineered strain of *C. necator* NSDG-GG, it was shown that higher concentrations of PHB can be produced from glucose by utilizing the RSM [55]. By using methanol as the sole source of carbon, the PHB production was successfully enhanced by the RSM using *Methylobacterium* sp. [56]. By using pineapple peel as the sole carbon source for the production of PHB by the *B. drentensis* strain BP17, the RSM was useful in improving the production of PHB by the strain [57]. According to Hassan et al. [58], the efficiency optimization of the PHB production by a novel *B. subtilis* strain from rice bran was achieved through the RSM with the Box–Behnken-design technique. Furthermore, the RSM improves the production of poly(3-hydroxybutyrate-co-3-hydroxyvalerate) (PHBV), which is a copolymer containing PHA molecules. In a recent study, it was discovered that the yeast strain *Wickerhamomyces anomalus* VIT-NN01 [59] is capable of producing PHBV using sugarcane molasses supplemented with other carbon sources, including palm oil and corn-steep liquor. In addition to this, the RSM has also been used as a tool to evaluate the optimum operating conditions for composites combining PHBV and tapioca starch [60].

Meanwhile, statistical analyses revealed a nonsignificant value of the lack of fit (*p* > 0.05), and a highly significant level of the model (*p* < 0.0001), implying that the mode could be accurately predicted by variation [61]. The current RSM result for increased PHB production using a low-cost agro-waste source would be advantageous for application and commercialization.

## 5. Conclusions

In this study, seven different bacterial strains were isolated from mangrove soil samples and screened for PHB production. The highest PHB production by the isolated *A. indicus* strain B2 was obtained after 96 h at a pH of 8.0, 35 °C, and 2% NaCl, with glucose and peptone as the carbon and nitrogen sources, respectively. A UV–Vis spectrophotometer and an FTIR analysis were used for the characterization of the extracted polymer to confirm its structure as a PHB, and the presence of the *phbB* gene was confirmed via PCR amplification. Various cheap agricultural wastes, including rice, barley, corn, and wheat bran, were efficiently utilized by the isolate *A. indicus* strain B2, which enabled it to produce PHB, among which rice bran was found to be the most efficient carbon source for PHB production. The RSM approach through the CCD for optimizing PHB synthesis was highly efficient at augmenting the polymer yield. Under optimum conditions, obtained from the RSM, this strain can improve the production of the PHB content by around 1.4-fold when compared with an unoptimized medium. Therefore, the RSM is a powerful tool for optimizing PHB production. In order to optimize PHB synthesis using the RSM through the CCD, a high efficiency was shown to augment the polymer yield through the use of this approach. The study provides valuable information about the optimal conditions for PHB production by *A. indicus*, which is a viable substitute for petroleum-based plastics in a variety of industrial applications. In order to achieve more economical commercial production under the conditions of fermenters, further studies will be needed.

## Figures and Tables

**Table 1:** Variables optimized by CCD for PHB production.

Name of Variable with Code	Unit	Range and Levels		
		−1	0	+1
Rice bran (A)	g/L	10	20	30
Peptone (B)	g/L	2.5	5	7.5
Temperature (C)	°C	30	35	40

**Table 2:** Central composite design along with experimental and predicted values of dependent variable for PHB production.

Run	A	B	C	PHB Production (g/L) ± SD	
				Experimental	Predicted
1	1	−1	1	1.58 ± 0.08	1.99
2	1	−1	−1	0.55 ± 0.06	1.11
3	0	−1.6817	0	4.17 ± 0.15	3.46
4	−1	−1	1	3.52 ± 0.08	3.78
5	1.6817	0	0	0.01 ± 0.01	−0.4603
6	0	0	0	5.57 ± 0.15	5.65
7	0	0	1.6817	4.44 ± 0.09	4.33
8	0	0	−1.6817	3.31 ± 0.1	3.21
9	−1	1	1	4.33 ± 0.1	3.91
10	−1.6817	0	0	3.35 ± 0.18	3.62
11	−1	−1	−1	4.35 ± 0.07	4.44
12	0	0	0	5.3 ± 0.05	5.65
13	0	0	0	6.11 ± 0.16	5.65
14	0	0	0	5.7 ± 0.1	5.65
15	1	1	−1	0.52 ± 0.05	0.4064
16	0	0	0	5.47 ± 0.160	5.65
17	0	1.6817	0	2.47 ± 0.05	2.98
18	0	0	0	5.72 ± 0.07	5.65
19	−1	1	−1	3.72 ± 0.08	3.46
20	1	1	1	2.34 ± 0.12	2.39

Experiment was carried out in triplicates; SD: standard deviation.

**Table 3:** ANOVA for PHB production as a function of independent variables.

Source	Sum of Squares	*df*	Mean Square	F-Value	*p*-Value Probe > *F*
**Model**	64.1	9	7.12	31.37	<0.0001 *
A: Rice bran	20.05	1	20.05	88.3	<0.0001 *
B: Peptone	0.2782	1	0.2782	1.23	0.2943
C: Temperature	1.5	1	1.5	6.62	0.0278 *
AB	0.0378	1	0.0378	0.1665	0.6918
AC	1.18	1	1.18	5.19	0.046 *
BC	0.6216	1	0.6216	2.74	0.129
A²	29.89	1	29.89	131.64	<0.0001 *
B²	10.67	1	10.67	46.98	<0.0001 *
C²	6.36	1	6.36	27.99	0.0004 *
**Residual**	2.27	10	0.2271		
Lack of Fit	1.89	5	0.3781	4.97	0.0515 ^ns^
Pure Error	0.3801	5	0.076		
**Cor Total**	66.37	19			

*df*: degree of freedom; ns: not significant; * significant.

## Data Availability

All data generated or analyzed during this study are included in this article.
